# Dual Doping of Silicon and Manganese in Hydroxyapatites: Physicochemical Properties and Preliminary Biological Studies

**DOI:** 10.3390/ma12162566

**Published:** 2019-08-12

**Authors:** Katarzyna Szurkowska, Agata Drobniewska, Joanna Kolmas

**Affiliations:** 1 Department of Analytical Chemistry and Biomaterials, Analytical Group, Faculty of Pharmacy with Laboratory Medicine Division, Medical University of Warsaw, ul. Banacha 1, 02-097 Warsaw, Poland; 2 Department of Environmental Health Sciences, Faculty of Pharmacy with Laboratory Medicine Division, Medical University of Warsaw, ul. Banacha 1, 02-097 Warsaw, Poland

**Keywords:** hydroxyapatite, manganese, silicon, biomaterials, infrared spectroscopy, nuclear magnetic resonance

## Abstract

Silicated hydroxyapatite powders enriched with small amounts of manganese (Mn^2+^) cations were synthesized via two different methods: precipitation in aqueous solution and the solid-state method. The source of Mn^2+^ ions was manganese acetate, while silicon was incorporated using two different reagents: silicon acetate and sodium metasilicate. Powder X-ray diffraction (PXRD) analysis showed that the powders obtained via the precipitation method consisted of single-phase nanocrystalline hydroxyapatite. In contrast, samples obtained via the solid-state method were heterogenous and contaminated with other phases, (i.e., calcium oxide, calcium hydroxide, and silicocarnotite) arising during thermal treatment. The transmission electron microscope (TEM) images showed powders obtained via the precipitation method were nanosized and elongated, while solid-state synthesis produced spherical microcrystals. The phase identification was complemented by Fourier transform infrared spectroscopy (FTIR). An in-depth analysis via solid-state nuclear magnetic resonance (ssNMR) was carried out, using phosphorus ^31^P single-pulse Bloch decay (BD) (^31^P BD) and cross-polarization (CP) experiments from protons to silicon-29 nuclei (^1^H → ^29^Si CP). The elemental measurements carried out using wavelength-dispersive X-ray fluorescence (WD-XRF) showed that the efficiency of introducing manganese and silicon ions was between 45% and 95%, depending on the synthesis method and the reagents. Preliminary biological tests on the bacteria *Allivibrio fisheri* (Microtox®) and the protozoan *Spirostomum ambiguum* (Spirotox) showed no toxic effect in any of the samples. The obtained materials may find potential application in regenerative medicine, bone implantology, and orthopedics as bone substitutes or implant coatings.

## 1. Introduction

Among the calcium phosphates, hydroxyapatite (HA) (Ca_10_(PO_4_)_6_(OH)_2_) has received considerable attention as a bone substitute material for orthopaedic and dental applications due to its strong affinity toward human mineralized tissues (bone and teeth) [1,2,3,4]. Biological apatite is nanocrystalline and contains various ions as impurities, mainly magnesium (Mg^2+^) and carbonates (CO_3_^2−^), with other trace elements in various amounts (Zn^2+^, K^+^, Na^+^, Mn^2+^, SiO_4_^4−^, Cl^−^, and F^−^) [5,6,7]. It should be noted that synthetic hydroxyapatites are highly susceptible to ionic substitution. Therefore, the introduction of various ions into the crystal structure of hydroxyapatite allows for obtaining material similar to bones and teeth. This is based on the concepts of biomimetics, where the greater the similarity existing between an implant and a tissue, the better they are expected to interact with each other [8,9]. It is important to note that ionic substitutions can change some physicochemical properties, such as the crystallographic lattice parameters, crystal morphology, and the degree of crystallinity. Substituting oxy-ions, (i.e., SiO_4_^4−^, CO_3_^2−^) and cations with various charges for the orthophosphates or structural hydroxyl groups and calcium ions, respectively, may lead to destabilization of the crystal lattice and a higher dissolution rate. Moreover, even a trace addition of some elements can significantly influence the thermal stability, solubility, or bioactivity of biomaterial, in vitro as well as in vivo [5,6,7].

Both manganese and silicon are classified as trace elements, with an average bone content of 1.7–3 ppm and 100–150 ppm, respectively [10,11]. The divalent manganese ion has the ability to activate integrins, a family of receptors that facilitate cellular adhesion [12]. Stimulation of bone cell adhesion, viability, and proliferation has a beneficial effect on the interaction of the implant with the host bone tissue [13,14]. Manganese is also a cofactor in enzymes, such as glycosyltransferases, involved in the remodelling of the extracellular matrix present in bone and cartilage [15]. Studies have shown that manganese supplementation in ovariectomized rats inhibits bone loss [16]. A key role for manganese in maintaining normal bone mass has been suggested, indicating its deficiency as a likely cause of osteoporosis [17,18]. Other mechanisms of manganese’s beneficial effects on bone tissue metabolism include osteocalcin stimulation, increased alkaline phosphatase activity and collagen type I production [13,14,19]. Syntheses of hydroxyapatites substituted with various amounts of manganese were carried out, from trace amounts (a few ppm) to 11.9 wt% [14,16,19,20,21,22,23,24]. However, it is important to note that in vitro studies on osteoblast cells showed significantly better results for lower manganese contents [16,19], while a Mn content of 1 wt% was regarded as high and caused drastically lower cell viability [24].

In contrast to manganese, silicon has a well-established position as a bioactivity-improving additive for bone biomaterials [11,25,26]. Carlisle’s landmark studies have defined the appropriate level of silicon required for the proper development and mineralization of osseous tissue [27,28]. The mechanism of silicon action is based on the positive impact of orthosilicic acid on the synthesis of type-I collagen [29,30]. Moreover, in the presence of orthosilicic acid in the medium, osteoblast-like MG-63 cells exhibit increased alkaline phosphatase activity and osteocalcin synthesis [29,30]. Furthermore, exposure to silicon has a stimulatory effect on the differentiation, proliferation, and activity of osteoblasts [31,32,33,34]. Thus, partial replacement of the hydroxyapatite phosphate group by silicate ions should further improve the biological activity of the bioceramic. Numerous syntheses of Si-substituted hydroxyapatite (Si-HA) were carried out, introducing up to 5 wt% of Si, which corresponds to the introduction of 1.7 moles of Si per mole of HA [35,36,37,38,39,40,41,42,43,44,45,46,47]. According to the research, to ensure phase uniformity of the obtained material along with optimal bioactivity, substitution should not exceed 1 mole of Si per mole of HA [36,37]. The beneficial effect of silicon substitution in HA has been demonstrated in numerous in vitro and in vivo experiments. High in vitro activity was confirmed by apatite precipitation when the samples were soaked in simulated body fluid (SBF), as well as in studies on osteoblasts, osteoclasts, and osteosarcoma cell cultures [37,38,39,40,41]. In vivo studies have shown faster bone remodelling in Si-HA samples, which additionally testifies to the osteoconductive properties of Si-HA [42,43,44,45,46,47].

The aim of the current study was to synthesize novel hydroxyapatite material, co-substituted with manganese (II) and silicate ions. Based on the previously quoted results [16,19,36,37], substitutions of 0.017 wt% of manganese and 1.98 wt% of silicon were planned for the current research. Synthesis was carried in two different ways: via precipitation and solid-state method. Moreover, two silicate sources were used, to compare the effectiveness of substitution for various synthesis conditions. This work focused on the structure and physico-chemical properties of the dual-doped hydroxyapatites, which were characterized using transmission electron microscopy (TEM), powder X-ray diffractometry (PXRD), Fourier transform infrared spectroscopy (FTIR,) solid-state nuclear magnetic resonance (ssNMR), and wavelength dispersive X-ray fluorescence (WD-XRF). Preliminary acute toxicity tests were also conducted. 

## 2. Materials and Methods 

### 2.1. Preparation of Samples

Synthesis of the materials was performed using two different methods (the precipitation method and the solid-state method) with two different sources of silicon (Na_2_SiO_3_ or Si(CH_3_COO)_4_). The planned samples, with nominal composition Ca_9.997_Mn_0.003_(PO_4_)_5.3_(SiO_4_)_0.7_(OH)_1.3_ and intended (Ca + Mn)/(P + Si) molar ratio of 1.67, are summarized in Table 1. Regardless of the silicon source, Si was introduced into hydroxyapatite in the form of orthosilicate SiO_4_^4−^ ions. In the case of metasilicate ions, hydrolysis occurred under the reaction conditions.

Precipitation synthesis was carried out at room temperature. Stoichiometric amounts of Ca(NO_3_)_2_∙4H_2_O and (CH_3_COO)_2_Mn were dissolved in 400 mL of distilled water in a flask under constant stirring. A Si-containing solution (Na_2_SiO_3_ or Si(CH_3_COO)_4_ dissolved in 50 mL of distilled water) and a P-containing solution ((NH_4_)_2_HPO_4_ dissolved in 50 mL of distilled water) were added dropwise to the Ca-Mn precursor solution using separate burettes. The pH was adjusted to approximately 10 using concentrated ammonia solution. The resultant precipitate was allowed to rest for 24 h for ageing. The precipitates were then filtered through a 0.8 µm pore size membrane filter under reduced pressure and rinsed several times with distilled water until the filtrate reached pH 7. This step was aimed at washing away residual reagents and soluble reaction products. The obtained precipitates were dried at 130 °C for 24 h and then ground in an agate mortar for physico-chemical and biological characterization.

For the solid-state synthesis, CaCO_3_, (CH_3_COO)_2_Mn, (NH_4_)_2_HPO_4_, and Na_2_SiO_3_ or Si(CH_3_COO)_4_ were used. The weighed substrates were ground in a ball mill and were pressed into tablets using a hydraulic press (pressure force 10 tons). The key step in the solid-state synthesis was sintering the tablets in the muffle furnace (Czylok FCF2,5SH, Jastrzębie-Zdrój, Poland) using the following temperature program: heating to 400 °C for 8 h, heating to 700 °C for 8 h, heating to 1000 °C for 8 h, then allowed to cool. To prevent the oxidation of Mn^2+^ ions, heating was carried out in an argon atmosphere. After the thermal treatment, the tablets were crushed in an agate mortar and the obtained powder was then subjected to further analysis.

### 2.2. Sample Characterization

The synthesized materials were analyzed via X-ray powder diffractometry (PXRD) using a Bruker D8 DISCOVER diffractometer (Bruker, Karlsruhe, Germany) with a Cu anticathode (λ = 1.54 Å). The scans were run from 20° to 70°, with a step size of 0.024°, a step time of 4 s, and a locked, coupled (theta–theta) geometry. Stoichiometric HA (inorganic crystal structure database ICSD #00-009-0432) was used as the reference pattern. On the basis of the diffraction patterns, the parameters of the unit cell (*a* and *c*) were estimated. The Scherrer equation (1) was used to calculate the crystal sizes of the samples synthesized by the precipitation method [48]:

(1) $$d=\frac{0.94\lambda }{\beta \cos\theta },$$

where

d—crystallite size (nm)
λ—radiation wavelength (nm)
β—the peak full width at half maximum intensity (radians)
θ—the diffraction angle of the corresponding reflex (°).

The reflections at approximately 25.8° and 39.9° were chosen for the calculation.

A JEOL JEM-1400 transmission electron microscope (TEM, Jeol LTD, Tokyo, Japan) with an accelerating voltage of 80 kV was used to observe the morphology of the obtained powders. In order to prepare the samples for TEM analysis, they were suspended in ethanol and then dropped on a copper grid covered with Formvar. 

Elemental analysis of the synthesized apatites was carried out via the WD-XRF method in the solid state, using tablets made of samples carefully triturated with microcrystalline cellulose. In order to determine the percentage weight of manganese and silicon in the samples, a standard curve of the signal-to-element concentration was made. The standard curve for each element consisted of five measurement points, while the blank sample was a tablet made of pure microcrystalline cellulose. To ensure uniform distribution of the standard in the tablet mass, the cellulose was mixed with a calculated amount of the standard solution, which was later evaporated. The cellulose was compressed into a tablet using a hydraulic press. Each measurement was carried out in triplicate using a WD-XRF ARL ADVANT’X spectrometer (Thermo Electron Corp., Waltham, MA, USA). The (Ca + Mn)/(P + Si) ratio of the samples was determined using energy-dispersive X-ray spectroscopic microanalysis (EDS INCA Energy TEM, Oxford Instruments, Abingdon, UK; Ca and P contents measured from six spots and then averaged). 

The chemical composition of the samples was investigated using Fourier transform infrared spectroscopy (FTIR, Perkin Elmer, Waltham, MA, US). The samples were ground with KBr in an agate mortar and then analyzed from 4000 to 400 cm^−1^ using a Perkin Elmer Spectrum 1000 spectrometer. Spectra were obtained within 30 repetitions (scans) with a resolution of 2 cm^−1^.

Solid-state NMR experiments on ^31^P and ^29^Si nuclei were performed using a Bruker WB 400 spectrometer (Bruker, Karlsruhe, Germany). The following experiments were carried out: ^31^P single-pulse Bloch decay (^31^P BD) and cross-polarization from protons to silicon-29 nuclei (^1^H → ^29^Si CP). The samples were spun at 7 and 5 kHz for the ^31^P and ^29^Si experiments, respectively, at the magic angle spinning (MAS), using ZrO_2_ rotors. The ^31^P and ^29^Si chemical shifts were referenced to external 85% orthophosphoric acid and tetramethylosilane (TMS), respectively.

Preliminary toxicity tests were performed using two tests: Microtox® and Spirotox. Two different suspensions were prepared, i.e., 1 and 2 mg/mL. The Microtox® test was based on the lyophilized luminescent bacteria *Allivibrio fischeri*. The suspended samples were incubated for 15 min with the bacteria in disposable glass cuvettes and then the light output of the samples was measured in the Microtox® M500 analyser (Osprey Scientific, Edmonton, AB, Canada). All samples were run in duplicate. For the control, 2% NaCl was used.

The protozoan *Spirostomum ambiguum* was used in the Spirotox test, which was performed in polystyrene multiwell plates according to the standard protocol [49]. In each well, 10 organisms were placed together with the suspended materials. The samples were incubated in the dark at 25 °C for 24 and 48 h. Then, a dissection microscope was used to analyze the lethal response or deformation of the *S. ambiguum* cell. The sample was tested in triplicate in each acute toxicity test (Microtox® and Spirotox); however, the tests were repeated (performed twice). Tyrode’s solution was used as a control.

## 3. Results and Discussion

### 3.1. PXRD, TEM, and Elemental Analysis Results

**Figure 1:**
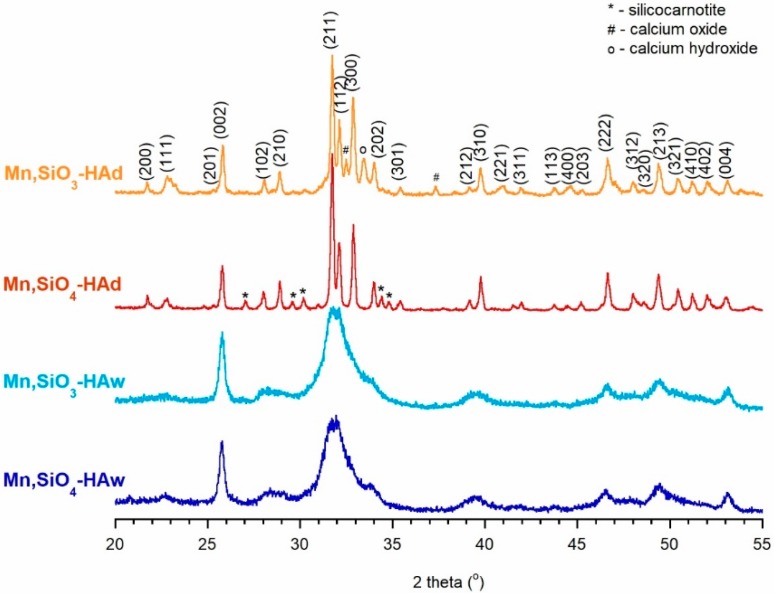
PXRD patterns of the analyzed samples.

The PXRD patterns of the powders synthesized by the precipitation method (Mn,SiO_3_-Haw, and Mn,SiO_4_-HAw) revealed the presence of poorly crystallized hydroxyapatite (see Figure 1). The reflections were very wide and weakly resolved, and some of them were too broad to be detected. Therefore, it may be suggested that the materials were nanocrystalline. The calculations based on the Scherrer equation allowed the crystal size to be estimated for the Mn,SiO_4_-HAw and Mn,SiO_3_-HAw nanocrystalline samples. The lengths of crystallites were 11 ± 2 nm and 13 ± 3 nm, while the widths were 6 ± 2 nm and 7 ± 2 nm, respectively.

In contrast, in the PXRD diffractograms of the samples prepared using the solid-state method (Mn,SiO_3_-HAd and Mn,SiO_4_-HAd), narrow and well-resolved reflections were observed (see Figure 1). It is worth noting that the solid-state method of synthesis led to extra reflections on the diffractogram patterns of both samples. In the Mn,SiO_3_-HAd pattern, the additional reflections at ca 32.45°, 37.45°, and 33.42° may be attributed to calcium oxide and calcium hydroxide, respectively [50,51]. The results of Rietveld refinement showed that the sample Mn,SiO_3_-HAd contained 3 wt% of CaO and 2 wt% of Ca(OH)_2_. On the other hand, the Mn,SiO_4_-HAd pattern presented additional reflections at approximately 34.7°, 30.2°, 29.6°, 26.9°, and 34.4° that originated mainly from silicocarnotite (Ca_5_(PO_4_)_2_SiO_4_) [51,52]. It should be noted that during calcination, in both cases, the obtained hydroxyapatites partially decomposed. This is usually promoted by non-stoichiometry and the presence of impurities or additives, i.e., substituted ions. Andreev et al. [51] suggested that silicocarnotite is a material produced during high-temperature calcination of calcium phosphates with silicon sources. According to the Rietveld analysis, the Mn,SiO_4_-HAd sample contained 5 wt% of silicocarnotite (JCPDS #40-393).

The parameters (*a* and *c*) of the apatitic crystal unit cell of the obtained samples (see Table 1) were found to be slightly different from those of stoichiometric HA (*a* = 9.432 Å and *c* = 6.881 Å), but it is difficult to find any regularities. According to the literature, the introduction of silicon ions should cause a decrease in the parameter *a* and an increase in the parameter *c*, while manganese substitution should reduce both parameters [5,6,7]. There was a distinct decrease in parameter *a* and an increase in parameter *c* in both of the solid-state-synthesized samples. On the other hand, the unit cell parameters of the samples obtained via the precipitation method did not change significantly.

**Figure 2:**
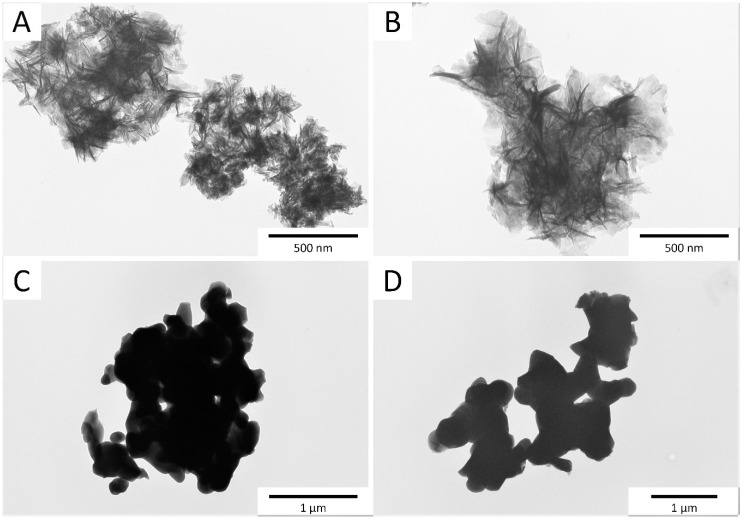
TEM images of the analyzed samples: (**A**) Mn,SiO_4_-HAw, (**B**) Mn,SiO_3_-HAw, (**C**) Mn,SiO_4_-Had, and (**D**) Mn,SiO_3_-HAd.

The TEM micrographs of the samples obtained via the precipitation method (Mn,SiO_4_-HAw and Mn,SiO_3_-HAw) are shown in Figure 2. The powders were nanosized, and the crystals were elongated and needle-shaped. This is in clear accordance with the literature where both the addition of manganese and silicate ions caused the formation of needle-like crystals [5,6,7]. Moreover, the crystals exhibited a high tendency to agglomerate, and therefore it was difficult to measure crystal sizes. However, we observed that the Mn,SiO_4_-HAw sample was characterized by smaller crystals compared to the Mn,SiO_3_-Haw sample. As shown in Figure 2, the crystals were heterogenous and differed significantly in size, while the calculation using the Scherrer equation shows the average crystal size. Synthesis in the solid state caused the formation of large crystal agglomerates, with sizes of up to approximately 0.5 μm. During the long heat treatment, the crystals formed a spherically shaped mould. As in the case of precipitation synthesis, the sample obtained from silicon acetate (Mn,SiO_4_-HAd) appeared to have smaller crystals. However, the difference was not as pronounced as in the precipitation method.

The Mn and Si content in each of the samples prepared using the two methods are presented in Table 1. In the Mn,SiO_3_-HAw and Mn,SiO_4_-HAw samples, manganese concentrations reached 50.6% and 64.7% of the nominal values, respectively. For the samples synthesized via the solid-state method, the Mn content was slightly lower (44.7%–47.1% of the nominal concentration). It is worth noting that substitution with Mn^2+^ ions was not trivial and ran with low efficiency. Recent works on the synthesis of hydroxyapatites enriched in manganese indicate that the substitution takes place within a limited range (up to 10 mol%) [22,24]. In addition, the thermal treatment of Mn-HA causes the decomposition of material at a fairly low temperature (600 °C) [21,24]. In contrast, the silicon substitution efficiency in Mn,SiO_3_-HAd and Mn,SiO_4_-HAd was significantly higher (91.9% and 94.9%, respectively) than in the samples produced using precipitation (61.5% and 76.4% of the nominal value for Mn,SiO_3_-HAw and Mn,SiO_4_-Haw, respectively). According to the literature, silicate ions can be located both inside the crystals (in the crystal’s core) and on the surface in the so-called hydrated surface layer. During the synthesis using the precipitation method, nanocrystals with an expanded surface layer were obtained [48]. Repeated washing of the obtained precipitates may lead to a partial loss of silicates, and thus lower substitution efficiency. In addition, silicates may “compete” with carbonates in the substitution of orthophosphates in the apatitic lattice. During the solid-state synthesis, carbonate ions contamination was easily removed by thermal decomposition. It is also worth mentioning that simultaneous substitution with silicate and manganese ions may be hindered due to the differences in ionic radii of Ca^2+^/Mn^2+^ (100 vs. 67 pm) and PO_4_^3−^/SiO_4_^4−^ (238 vs. 240 pm). Verification of such a hypothesis requires further research (especially PXRD detailed analysis).

### 3.2. FTIR Spectroscopy

**Figure 3:**
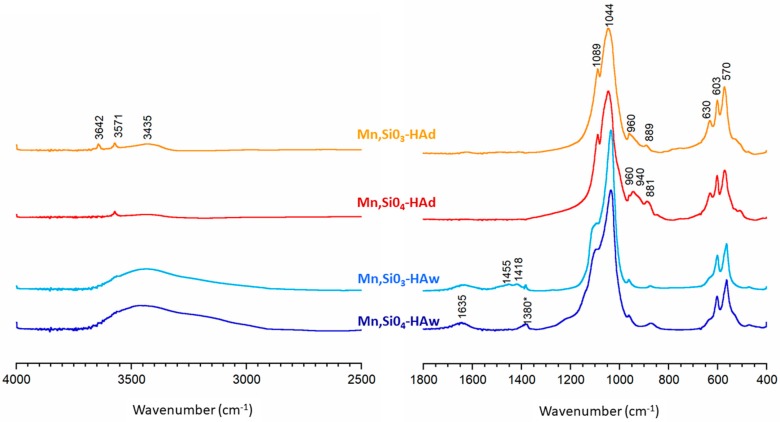
FTIR transmission spectra of the analyzed samples.

The FTIR spectra of the synthesized samples are shown in Figure 3. The absorption stretching bands in the region 1200–960 cm^−1^ may be assigned to the typical orthophosphate vibrations for hydroxyapatites. Phosphate bending bands (ν_4_) were observed at 570 and 603 cm^−1^ [53,54,55,56,57]. A weak band of structural hydroxyl groups at 3570 cm^−1^ was clearly detectable in the Mn,SiO_3_-HAd and Mn,SiO_4_-HAd spectra, while in the Mn,SiO_3_-HAw and Mn,SiO_4_-HAw spectra, it was partially obscured by a broad band in the 3700–2500 cm^−1^ region. This broad band and the band at 1640 cm^−1^ were due to moisture in the samples synthesized via the precipitation method, and they corresponded to the stretching and bending vibrations of water, respectively. As expected, in the spectra of the samples obtained via the solid-state method, the bands from water were very weak, whereas the band located at 630 cm^−1^, corresponding to librational vibration of structural hydroxyl groups, was easily detectable [53,54,55,56,57]. In addition, weak bands at 1455 and 1418 cm^−1^ observed in the Mn,SiO_3_-HAw spectrum, was attributed to carbonates (types A + B and B, respectively) [54].

It should be noted that during the synthesis, carbonates may be easily substituted for hydroxyl groups (type A) and orthophosphates (type B) [5,6,7]. The presence of carbonate bands was consistent with the lowest silicon content in this sample, since carbonates and orthosilicate ions compete for the same sites in the apatite structure. Moreover, in the spectrum of the Mn,SiO_3_-HAd sample, a narrow and weak band at 3642 cm^−1^ was probably due to the OH groups of calcium hydroxide, which, according to the PXRD pattern, represented a secondary phase present after synthesis via the solid-state method. Spectra for both the samples obtained using the solid-state method contained additional bands in the 940–840 cm^−1^ region. These were consistent with the literature and can be attributed to silicates [35,36,37,58,59]. The band located at approximately 880–890 cm^−1^, visible in all samples, can be assigned to Si-O vibration modes of SiO_4_^4−^ tetrahedra groups, while a band at 940 cm^−1^ in the Mn,SiO_4_-HAd sample corresponds to vibrations of the Si-OH bond. Indirect evidence for the substitution of orthosilicate ions into hydroxyapatite was observed as low intensity hydroxyl bands at approximately 3570 cm^−1^ and 630 cm^−1^. According to the reaction mechanism proposed by Gibson et al. [35], substitution of the trivalent orthophosphate group by a tetravalent orthosilicate group results in the loss of a hydroxyl group in order to maintain the charge balance. It is worth noting that the complex group of silicate bands is diminutive in the spectra of the Mn,SiO_4_-HAw and Mn,SiO_3_-HAw samples. This can be explained by the lower silicon content and the lower degree of crystallinity of the precipitated samples. In addition, the silicate bands may be obscured by signals from phosphates and carbonates occurring at similar frequencies [54,56].

### 3.3. ^31^P and ^29^Si MAS NMR Spectroscopy

**Figure 4:**
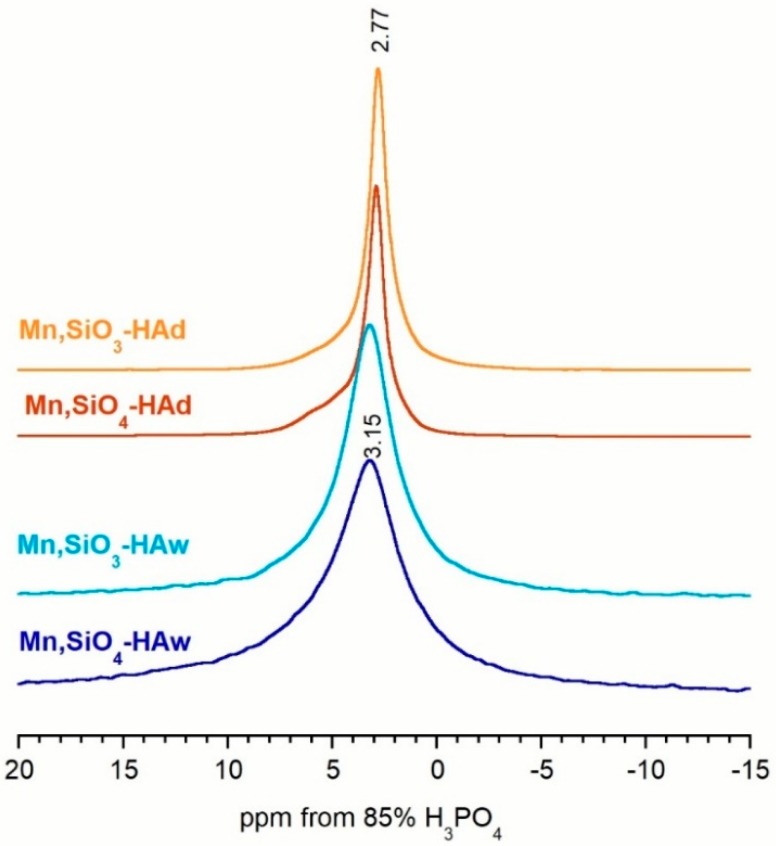
^31^P BD NMR spectra of the analyzed samples.

**Figure 5:**
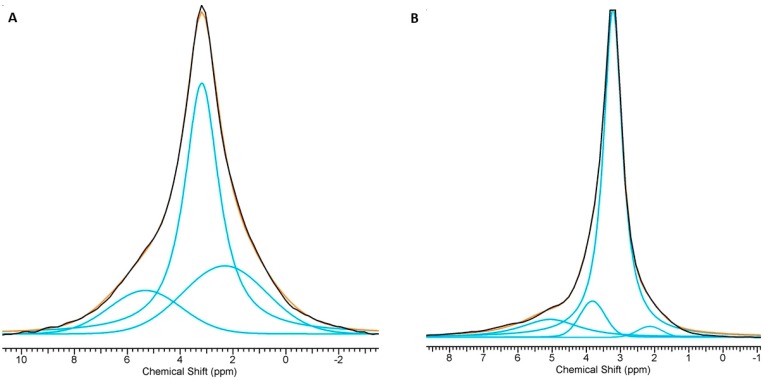
Representative deconvolution of the ^31^P BD NMR spectra: (**A**) Mn,SiO_4_-Haw and (**B**) Mn,SiO_4_-HAd.

The phosphorus-31 resonance spectra of all the powders acquired using the BD technique are shown in Figure 4. As expected, all the spectra exhibited a quite narrow signal at about 2.75–3.15 ppm, which according to the literature, can be attributed to orthophosphate groups located in well-ordered apatitic crystals [60,61,62]. It should be noted that the lines obtained from the powders synthesized using precipitation were significantly wider than those obtained from the solid-state-synthesized samples. Moreover, all obtained spectra are slightly asymmetric, which suggests the presence of additional signals. Deconvolution revealed additional lines for all the spectra (see Figure 5 for examples and Table 2). In the Mn,SiO_4_-HAw and Mn,SiO_3_-HAw samples, apart from the main signal, two additional signals were detected: one at ≈2.30 ppm and another at ≈5.30 ppm. According to the literature, these additional lines come from the disordered structure of the hydrated surface layer [60,62]. The signals at ≈2.14–2.33 ppm and ≈5.10–5.35 ppm detected in all the spectra may be assigned to protonated (≡PO_x_H) and unprotonated (≡PO_x_) surface groups (where ≡ stands for a link to the crystal surface), respectively [60,61,62]. Due to overlap of the ^31^P NMR lines, it is not straightforward to determine the contribution of ^31^P nuclei from the disordered phase to the total ^31^P reservoir. However, based on the deconvolution of the spectra, it can be estimated that in the Mn,SiO_3_-HAw and Mn,SiO_4_-Haw samples, 42.5%–45.5% of the phosphorus-31 nuclei come from ^31^P nuclei from the disordered phase.

As presented in Figure 5 and Table 2, the Mn,SiO_4_-HAd and Mn,SiO_3_-HAd spectra revealed three additional lines. Apart from the main signal at 3.15–3.22 ppm and the weak and broad lines (from protonated (≡PO_x_H) surface groups and from unprotonated (≡PO_x_) surface groups), an additional line at ≈3.85–4.12 ppm was easily detected. Its contribution is not very significant (7.5% and 2.1% for Mn,SiO_4_-HAd and Mn,SiO_3_-HAd samples, respectively). It can probably be assigned to the silicocarnotite phase [51]. The PXRD pattern of Mn,SiO_3_-HAd did not contain reflections from silicocarnotite, but this may be explained by the small amount of this crystalline phase. Its presence in the Mn,SiO_3_-HAd sample was confirmed using a ^31^P BD NMR experiment.

It should be noted that the resonance lines originating from the phosphorus-31 nuclei in the disordered phase (especially in the hydrated surface layer) were relatively weak (up to 16.4%), suggesting that the –POH groups at the surface were relatively sparse. This is in accordance with the presumption that the hydrated surface layer of apatitic crystals synthesized under high-temperature conditions (via the solid-state method) is exiguous.

**Figure 6:**
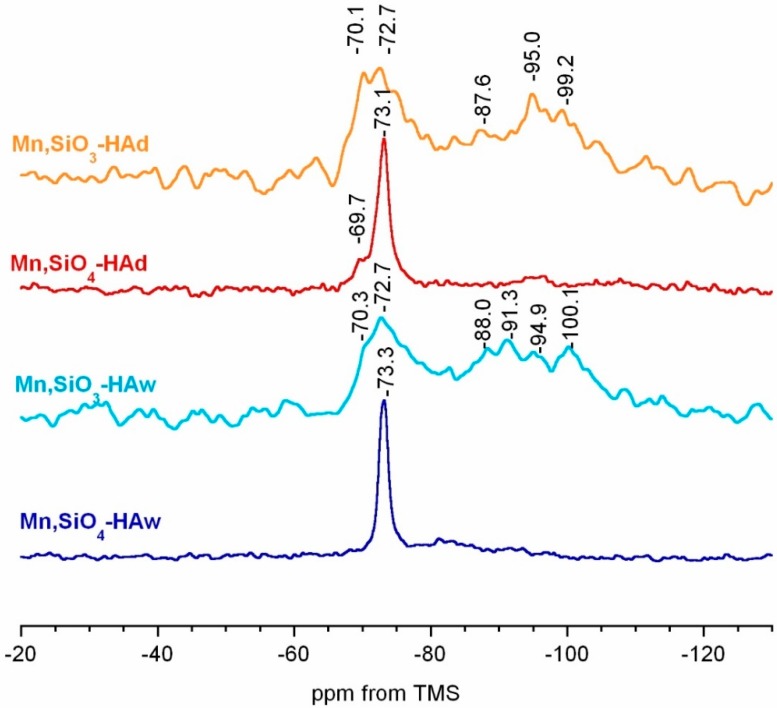
^29^Si CP MAS NMR spectra of the analyzed samples.

The standard ^1^H → ^29^Si CP MAS spectra are presented in Figure 6. Because of the low content of Si in the materials, the spectra presented significant noise, despite the long accumulation time. In all the spectra, a resonance line at approximately −72.7 to −73.3 ppm was predominant and can be assigned to a Q^0^ structure of silicates located within the crystal core [63,64,65,66]. It should be noted that in the Mn,SiO_4_-HAw spectrum, only one signal (−73.3 ppm) could be detected. However, in the Mn,SiO_3_-HAd, Mn,SiO_3_-Haw, and Mn,SiO_4_-HAd spectra, the resonance in this region was very broad and a second line (at approximately −69.3 to −70.3 ppm) was clearly visible. It could possibly be associated with the Q^0^ silicates located at the crystal surface. Surprisingly, in the spectra of the Mn,SiO_3_-HAw and Mn,SiO_3_-HAd samples, multiple broad and poorly resolved lines occurred in the region −88.0 to −100.1 ppm. According to the literature, these correspond to Q^3^/Q^4^ and are characteristic of non-crystalline silica species [36,64]. Therefore, we can conclude that the introduction of silicates into the apatitic crystal lattice seems to be more limited with sodium metasilicate as a source than with silicon acetate.

### 3.4. Preliminary Biological Tests

In order to evaluate the acute toxicity, the luminescent bacterium *A. fisheri* (Microtox®) and protozoan *S. ambiguum* (Spirotox) tests were used. Although manganese and silicon are essential trace elements, it was planned to check the toxicity of the Mn and Si content in the nanocrystals (Mn,SiO_4_-HAw and Mn,SiO_3_-HAw) and microcrystals (see Table 3). The obtained samples were observed to be non-toxic in both tests. These preliminary studies showed that the silicon and manganese contents of 1.22–1.88 wt% and 0.0076–0.0110 wt%, respectively, in HAs were completely neutral, and the obtained powders can be treated as promising materials for future biological investigations, i.e., cytotoxicity and biocompatibility.

## 4. Conclusions

Novel manganese and silicate co-substituted apatite materials were synthesized in this study. The study was aimed at comparing the physico-chemical properties of samples depending on the reagent used and the synthesis method. Samples were characterized using PXRD, FTIR, and ssNMR spectroscopy, as well as TEM and WD-XRF. Preliminary biological in vitro tests were also performed. Significant conclusions from these examinations are presented below.

1. Samples obtained using precipitation were nanocrystalline and monophasic. Solid-state samples were microcrystalline and contained secondary phases (impurities), i.e., calcium oxide, calcium hydroxide, and silicocarnotite, which were produced during the sintering process.
2. Manganese and silicon ions were successfully incorporated, as confirmed using WD-XRF and spectroscopic measurements. The substitution efficiency was between 45% and 95%, depending on the synthesis method and the reagents.
3. A higher efficiency of Mn^2+^ substitution occurred in the case of precipitation synthesis, while the substitution of silicon was favoured in the solid-state synthesis. In both cases, the yield was better when using silicon acetate instead of sodium metasilicate as a silicon source.
4. The introduction of ions did not significantly affect the crystallinity and unit cell parameters. The degree of crystallinity, as well as the size and shape of the crystals, depended mainly on the synthesis method.
5. According to the Microtox® and Spirotox tests, none of the samples was considered to be toxic. Such promising results may constitute the starting point for further biological research.

## Figures and Tables

**Table 1:** Various parameters of the obtained samples.

	Mn,SiO_3_-HAw	Mn,SiO_4_-HAw	Mn,SiO_3_-HAd	Mn,SiO_4_-HAd
Synthesis method	Precipitation		Solid-state	
Silicon source	Na_2_SiO_3_	Si(CH_3_COO)_4_	Na_2_SiO_3_	Si(CH_3_COO)_4_
Parameter *a* (Å) ^a^	9.424	9.433	9.418	9.415
Parameter *c* (Å) ^a^	6.882	6.875	6.886	6.896
Mn content (wt%)	0.0086 ± 0.0004	0.0110 ± 0.0004	0.0076 ± 0.0003	0.0080 ± 0.0004
Si content (wt%)	1.217 ± 0.005	1.512 ± 0.003	1.819 ± 0.003	1.879 ± 0.004
Mn substitution efficiency (%)	50.59	64.71	44.71	47.06
Si substitution efficiency (%)	61.48	76.34	91.88	94.90
(Ca + Mn)/(P + Si) molar ratio	1.54 ± 0.05	1.59 ± 0.04	1.61 ± 0.03	1.62 ± 0.05

^a^ Error was ±0.3%.

**Table 2:** NMR deconvolution results for the ^31^P BD NMR experiments (FWHM—full width at half maximum).

	Assignments	Chemical Shift (ppm)	FWHM (Hz)	Percentage of Total Area (%)
Mn,SiO_4_-HAw	≡POxH	2.31	606	27.4
	main	3.18	256	57.4
	≡POx	5.31	523	15.1
Mn,SiO_3_-HAw	≡POxH	2.25	640	22.3
	main	3.22	248	54.5
	≡POx	5.28	551	23.2
Mn,SiO_4_-HAd	≡POxH	2.14	138	2.4
	main	3.22	108	77.0
	≡POx	5.10	329	12.1
	silicocarnotite	3.85	142	7.5
Mn,SiO_3_-HAd	≡POxH	2.33	145	4.2
	main	3.15	111	78.5
	≡POx	5.35	316	15.2
	silicocarnotite	4.12	156	2.1

**Table 3:** Toxicity of the synthesized powders in Microtox® and Spirotox tests.

Sample	Microtox® (15 min-PE) ^1^		Spirotox ^2^	
	1.0 mg/mL	2.0 mg/mL	1.0 mg/mL	2.0 mg/mL
Mn,SiO_4_-HAw	0	0	NT	NT
Mn,SiO_3_-HAw	0	0	NT	NT
Mn,SiO_4_-HAd	0	0	NT	NT
Mn,SiO_3_-HAd	0	0	NT	NT

^1^ Percent of toxic effect after 15 min of incubation; ^2^ NT—not toxic.
